# The impact of dengue illness on social distancing and caregiving behavior

**DOI:** 10.1371/journal.pntd.0009614

**Published:** 2021-07-19

**Authors:** Kathryn L. Schaber, Amy C. Morrison, William H. Elson, Helvio Astete-Vega, Jhonny J. Córdova-López, Esther Jennifer Ríos López, W. Lorena Quiroz Flores, Alfonso S. Vizcarra Santillan, Thomas W. Scott, Lance A. Waller, Uriel Kitron, Christopher M. Barker, T. Alex Perkins, Alan L. Rothman, Gonzalo M. Vazquez-Prokopec, John P. Elder, Valerie A. Paz-Soldan

**Affiliations:** 1 Program of Population Biology, Ecology and Evolution, Emory University, Atlanta, Georgia, United States of America; 2 Department of Virology and Emerging Infections, U.S. Naval Medical Research Unit No. 6, Lima and Iquitos, Peru; 3 Department of Pathology, Microbiology, and Immunology, School of Veterinary Medicine, University of California, Davis, California, United States of America; 4 Department of Entomology and Nematology, University of California, Davis, California, United States of America; 5 Department of Biostatistics and Bioinformatics, Rollins School of Public Health, Emory University, Atlanta, Georgia, United States of America; 6 Department of Environmental Sciences, Emory University, Atlanta, Georgia, United States of America; 7 Department of Biological Sciences and Eck Institute for Global Health, University of Notre Dame, Notre Dame, Indiana, United States of America; 8 Institute for Immunology and Informatics and Department of Cell and Molecular Biology, University of Rhode Island, Providence, Rhode Island, United States of America; 9 School of Public Health, San Diego State University, San Diego, California, United States of America; 10 Department of Global Community Health and Behavioral Sciences, Tulane School of Public Health and Tropical Medicine, New Orleans, Louisiana, United States of America; Universidad de Buenos Aires, ARGENTINA

## Abstract

**Background:**

Human mobility among residential locations can drive dengue virus (DENV) transmission dynamics. Recently, it was shown that individuals with symptomatic DENV infection exhibit significant changes in their mobility patterns, spending more time at home during illness. This change in mobility is predicted to increase the risk of acquiring infection for those living with or visiting the ill individual. It has yet to be considered, however, whether social contacts are also changing their mobility, either by socially distancing themselves from the infectious individual or increasing contact to help care for them. Social, or physical, distancing and caregiving could have diverse yet important impacts on DENV transmission dynamics; therefore, it is necessary to better understand the nature and frequency of these behaviors including their effect on mobility.

**Methodology and principal findings:**

Through community-based febrile illness surveillance and RT-PCR infection confirmation, 67 DENV positive (DENV+) residents were identified in the city of Iquitos, Peru. Using retrospective interviews, data were collected on visitors and home-based care received during the illness. While 15% of participants lost visitors during their illness, 22% gained visitors; overall, 32% of all individuals (particularly females) received visitors while symptomatic. Caregiving was common (90%), particularly caring by housemates (91%) and caring for children (98%). Twenty-eight percent of caregivers changed their behavior enough to have their work (and, likely, mobility patterns) affected. This was significantly more likely when caring for individuals with low “health-related quality of well-being” during illness (Fisher’s Exact, p = 0.01).

**Conclusions/Significance:**

Our study demonstrates that social contacts of individuals with dengue modify their patterns of visitation and caregiving. The observed mobility changes could impact a susceptible individual’s exposure to virus or a presymptomatic/clinically inapparent individual’s contribution to onward transmission. Accounting for changes in social contact mobility is imperative in order to get a more accurate understanding of DENV transmission.

## Author summary

Dengue is the most important mosquito-borne viral disease of humans worldwide. Due to the limited mobility of the mosquitoes that transmit dengue virus, human mobility is a key variable to understanding the spread of dengue through a population. Recently it was shown that individuals with symptomatic dengue have significantly reduced mobility patterns. To better understand how dengue illness affects the behavior of visitors and caregivers, we examined the nature and frequency of a symptomatic individual’s social contacts to determine if their behaviors changed due to social distancing or caregiving. While many participants had a drop off in visitor frequency when ill, almost all participants received help from their housemates. These caregivers were most likely to have their work impacted when helping participants whose quality of life was most negatively affected by illness. We quantified how often these behavioral changes had a discernable effect on the social contact’s mobility patterns. Accounting for mobility changes by social contacts provides a more accurate understanding of infection risk and potential for virus spread through a population. Dengue transmission models that incorporate mobility changes of symptomatic individuals and their social contacts will add currently missing epidemiologically relevant detail for evaluating different disease prevention strategies.

## Introduction

Dengue, an acute illness caused by any of the four closely related dengue viruses (DENV), is the most important mosquito-borne viral disease of humans [1]. Due to the sedentary, day-biting behavior of the primary vector, *Aedes aegypti (A*. *aegypti)*, and its propensity for residential locations [2–5], human mobility and visitation patterns among residential locations shape human-mosquito contacts and DENV transmission dynamics [6–11]. Indeed, an individual’s risk of DENV infection increases when they routinely visit the same residential locations as other DENV-infected people [12]. Recently, studies have shown that individuals with symptomatic DENV infection have significant changes in their mobility patterns during illness, spending more time at home and visiting fewer locations during the first six days of illness [13,14]. These disease-driven mobility changes are predicted to lead to a large proportion of primary infectious bites occurring at the home of an infectious individual, which increases infection risk for people living in or visiting the residence [15]. Previous studies have demonstrated clustering of dengue cases at the household level, where the housemates of an index case have high infection incidence compared to those living further away [16–20].

An unresolved question is whether the social contacts of symptomatic dengue individuals (i.e., those who are *routinely* part of their social circle) also change their mobility and, if so, what the possible impacts could be for overall transmission dynamics. Many models of infections that are directly transmitted between humans account for social contacts changing their behavior with social distancing, where contact stops between the two individuals until the symptomatic period is over [21–24]. Social distancing has been shown to have a large impact on the spread of directly transmitted diseases, with its efficacy often dependent on the percent of the population practicing the behavior [21,25–27]. Despite its importance for directly transmitted diseases, social distancing has not yet been examined in the case of vector-borne diseases (VBDs). Moreover, even directly transmitted disease models rarely account for social contacts acting as caregivers, who are likely to increase their contact with symptomatic individuals [28]. Indeed, research on contacts providing health and social support has a long and rich history in the social sciences [29–33]; however, most of the literature emphasizes mental health and chronic disease. Social support for an ill person has four main functions: emotional support and empathy, instrumental support (aid and assistance), information (e.g. how to recover, where to find care), and appraisal (e.g. feedback on apparent severity and recovery) [34]. This support may derive from social norms that compel an individual to visit and care for a sick friend or relative, but it may also be given with the expectation of reciprocity if and when they themselves become sick [33]. While the role of social support has been examined for HIV/AIDS [35,36], there remains a large gap in the social support literature for other infectious diseases. Empirical information is needed both on the prevalence of social distancing from symptomatic dengue cases and the dynamics of caregiving behavior, particularly whether caregiving behavior affects mobility of social contacts.

Capitalizing on an established contact-cluster design [12], we solicited information on the visitors received by symptomatic DENV-infected individuals throughout their illness period and the home-based care received from housemates. In particular, we examined whether the caregiving behavior of social contacts affected their work, utilizing this as a proxy for mobility change. Comparisons of visitors received pre-illness and during illness informed the prevalence of mobility changes that could be attributed to social distancing or increased visitation. Given the endemic nature of dengue illness in our study site, we hypothesized that social distancing behaviors would only be moderately common. Comparatively, we predicted that caregiving behavior was common, with the majority of caregivers being adult housemates of the sick individual. We further hypothesized that the impact on the caregiver’s work would depend on the quality of life of the DENV-infected individual and the relation of their caregiver.

## Methods

### Ethics statement

This study was approved by the Institutional Review Board (IRB) of the United States Naval Medical Research Center Unit No. 6 (NAMRU-6) (Protocol #: NAMRU6.2014.0028) in compliance with all applicable federal regulations governing the protection of human subjects. IRB relying agreements were established between NAMRU-6 and Emory University, Tulane University, University of California Davis, University of Rhode Island, San Diego State University, and University of Notre Dame. In addition to IRB approval, investigators obtained host country approval from the Loreto Regional Health Department, which oversees health research in Iquitos. Adult study participants provided written informed consent and a parent or guardian provided informed consent on behalf of child study participants. Children 8–17 years of age provided written assent. The proposed interview schedule was discussed with each participant during the consent process. Study staff always accommodated participant schedules, made every effort to minimize the time needed for the interviews and clinical evaluation, and it was strongly emphasized that participation was voluntary and could be declined at any time. In practice, participants were unavailable on some occasions, but our research group has had a long term relationship with the community in Iquitos and visits by staff during illness are usually valued heavily by our participants. If our physician had any concerns about the severity of illness, they would help facilitate admission into a local hospital, and would continue visits only if requested by participants, which was often the case.

### Study area

This study was performed in the Amazon basin city of Iquitos, Peru. Iquitos is a geographically isolated, tropical urban environment with approximately 430,000 inhabitants located near the confluence of the Amazon, Nanay, and Itaya Rivers [37]. The city’s economic structure is highly informal and dynamic, with one-third of economically active individuals either unemployed or informally employed [38]. Iquitos has a fairly homogeneous mestizo culture, with the majority of residents being categorized in socio-economic levels C and D, comparable to middle- and lower middle-class.

Iquitos has been the home of extensive, long-term arboviral research led by the University of California, Davis and U.S. Naval Medical Research Unit No. 6 since 1998 [11,12,39–43]. Human mobility studies paired with detailed epidemiological data make Iquitos an informative site for understanding the dynamics of arbovirus transmission. All four serotypes of DENV have been introduced in Iquitos; however, at any particular time virus transmission is usually dominated by a single serotype [42,44]. Mobility is highly irregular and temporally unstructured, rarely centering around a single location, such as a workplace [40]. Previous research [40] demonstrated that the majority of individual’s movement (~80%) occurs within 1 km of their home.

### Study design

Iquitos residents with laboratory-confirmed DENV infection (by polymerase chain reaction [PCR]) were identified and recruited through clinic- and community-based longitudinal febrile surveillance as previously described [12]. At the time of initial diagnostic blood draw, a retrospective semi-structured movement survey (RMS) was verbally administered by trained research assistants (the ‘Movement Team’) to identify locations an individual had visited in the 15 days prior to diagnosis (the exposure period), as well as the visitors they received at their home in the previous three days. For the next seven days a modified survey that focused on activities and personal contacts during the prior 24 hour period (Daily RMS [DRMS]) was carried out [14]. During that seven-day follow-up period, DENV positive (DENV+) individuals were also administered two Quality of Well-Being (QWB) surveys [45], 2–3 days and seven days after the initial PCR-positive blood test result. The QWB survey is a validated instrument used to measure an individual’s health related quality of life during chronic illness [45,46] that uses a weighted algorithm to produce a well-being score between 0 (death) and 1 (optimal health) [45]. On the seventh day after the initial diagnostic blood draw an expenses survey was administered, which focused on the costs incurred during dengue illness. This survey had a subset of questions focusing on the general caregiving behaviors received by a symptomatic participant during his/her illness.

During a follow-up visit, scheduled 30 days after the initial PCR-positive blood test, participants were given “post-illness” RMS and QWB surveys in an effort to measure a baseline for their mobility behavior and well-being in the absence of illness. A second expenses survey was also administered in order to identify costs accrued after the initial day-7 survey. S1 Table provides a description of each survey, including when it was administered, the number of respondents, and a list of the questions that contributed to the current analysis.

### Data processing

For each study participant a standardized “day after symptom onset” variable was calculated, rather than referring to daily survey values as occurring on a certain number of days after study enrollment. For example, if a participant provided a blood sample on their third day of symptoms, daily surveys would capture data for days 4–11 after symptom onset. Because DENV cases were captured 1–5 days after onset of their symptoms, daily surveys captured a range of 1–14 days after symptom onset. We focused our analysis on days 1–10 after symptom onset, as only a few individuals (12/71) had data for days 11–14.

For each day after symptom onset, DRMS data were utilized to record (1) how many visitors an individual received at their home, (2) the visitor’s relation to the ill participant, (3) the reason for the visit, (4) if the visitor knew the person was sick at the time of visit, and (5) whether the visitor typically visited the participant at least once a week in the absence of illness (‘routineness’ of the visitor). All data were reported by the subject or his/her parent or guardian. Pre- and post-illness RMS provided a baseline for the number of visitors an individual received.

The ‘Expenses’ survey was given twice, with both datapoints focused on the entire illness period. The datapoints were crosschecked to ensure all caregiving instances were accounted for. Information was provided on (1) whether an individual had someone help care for them (i.e., act as a caregiver), (2) how many people helped, (3) the relation of each helper to the participant, (4) what they helped with, and (5) whether the helper had to take time off from work (i.e., was their work affected). Similarly, the QWB survey was given twice during the symptomatic period. The two datapoints were combined to determine whether individuals felt at any point during their illness that they *needed help* with daily activities or personal care. An individual’s QWB score, calculated with the weighted algorithm, was also provided for these two time points. An individual’s minimum QWB score across the two time points was utilized as a metric of overall health-related quality of life during illness. Minimum QWB score was considered as a continuous variable as well as a factor variable, where individuals were split into 2 (low/high) or 3 (low/medium/high) groups of equal size based on their minimum QWB score during illness.

### Data analysis

Our main goal of data analysis was to examine behavioral changes among social contacts in response to symptomatic dengue illness. Data on visitors and caregivers provided information on a symptomatic individual’s social contacts from inside their house and outside their house.

Details about visits received during the illness period were analyzed using the following variables: (1) presence of visitors (yes/no), (2) number of unique visitors (1/2+), (3) relation of visitors (family/friend/other), (4) number of unique visits by each visitor (1/2+), (5) whether they were ‘routine visitors’ (yes/no), (6) whether they knew that the person they came to visit was ill (yes/no), and (7) the reason for each visit (emotional support/logistic support/other disease reason/unrelated to disease). The first two variables were analyzed at the participant level, whereas (3)-(7) were analyzed at the visitor level. For each variable of interest, descriptive statistics were calculated and comparisons were made based on the gender (male/female) and age (adult:> = 18/child:<18) of the symptomatic individual, the gender/age combination (male adult/male child/female adult/female child), and the health-related quality of life (low/high minimum QWB score during illness). The reason for visiting was also compared based on whether the visitor was ‘routine’ or not. All these comparisons were conducted using Fisher’s Exact test. For non-dichotomous factor variables, (3) and (7), if there was a significant association with gender/age/quality of life during illness/’routineness’, the proportions for each outcome level were compared separately to determine if a certain outcome level was driving the association. Comparisons were also made between presence of visitors pre-illness and during illness to determine the prevalence of losing, gaining, or keeping visitors when ill.

Comparisons were conducted to determine whether the infected person’s gender, age, or quality of life during illness was associated with caregiving behaviors. Details about caregiving were captured for the overall illness period using the following variables: (1) presence of caregivers (yes/no), (2) number of caregivers (1/2), (3) whether the caregiver was a housemate of the sick individual (yes/no), (4) days help was provided, (5) whether the caregiver helped take care of the sick individual (yes/no), (6) whether the caregiver helped around the house (yes/no), (7) whether the caregiver helped by providing money or items (yes/no), and (8) whether their work was negatively affected due to providing help to the sick person (yes/no). Variables (1) and (2) were examined at the participant level and variables (3)-(8) were analyzed at the helper level.

For variables related to caregiving, the association with possible predictor variables was determined using Generalized Linear Models (GLMs). Best-fit models were determined for the logistic response variables of: presence of caregiving (yes/no), number of caregivers (one versus two), relationship of caregiver (housemate or not), if they helped around the house (yes/no), if they helped with buying things or giving money (yes/no), and if the caregiver’s work was affected (yes/no). For each response variable, individual predictor variables considered were the age (child vs. adult) and gender (male vs. female) of the sick individual (as well as an interaction variable for age and gender), whether the sick individual had a high or low number of housemates (split into a binary variable around the median number of housemates), whether they needed help with personal care activities or daily activities at any point during illness (QWB), and minimum QWB score (as a continuous value or using 2/3 groups). If two bivariate regressions had significantly good fits, a model with both variables was considered. Best-fit models were determined using the corrected AIC (AICc), relative likelihood of the model (weight), and a Chi-squared test comparing reduction in residual deviance.

The response variable of whether or not a symptomatic individual received visitors was examined for both the entire illness period and each day of illness. When the entire illness period was considered, GLMs were examined with the same predictor variables as above. When the response variable was the presence of visitors on any given day of illness, Generalized Linear Mixed Models (GLMMs) were used, with the participant ID as a random effect to account for repeated observations [47]. Specific day of illness was considered as a possible predictor variable to determine whether visitor presence changes throughout illness. Variables with set values that did not change during illness (i.e., gender, age, number of housemates, whether help was needed to complete daily or personal care activities, minimum QWB score) were also considered as possible predictors. All statistical analyses were performed in R 3.3.0 statistical computing software [47,48]. Note that multiple correspondence analysis was used to create an SES (socioeconomic) score based on an individual’s employment, education, and home materials (i.e., roof, floor, interior/exterior walls), as well as the presence of household items/services such as computers, “mototaxis” (rickshaws), cable, and internet. This score was stratified into three groups to compare outcomes; however, there were no significant differences found between groups and SES score was not a significant predictor variable in any of the GLM(M)s. This is likely because, outside of the more touristic areas of the city where one might find a higher SES, the SES status of Iquitos’ residents, specifically those in our study areas, is fairly homogeneous: middle to lower-middle class. Based on the possibly small margin between groups and the lack of significance on any outcome variables, we chose not to include the SES score in the results.

## Results

Detailed data were collected from 71 DENV+ participants about daily visitors received, 70 of whom provided QWB data, and 67 of whom provided caregiving data. Of the 67 participants with both datasets, 59.7% were children and 49.3% were male. Twenty of 27 adults worked, but 60% of those individual’s classified their work schedules as ‘flexible’. The majority of participants reported having an illness lasting five or more days according to the ‘Expenses’ survey (77%). During the illness period, 53% of individuals reported needing help with daily activities and 14% reported needing help with personal care. The minimum QWB score (i.e., the measurement when most ill) for participants was normally distributed with an average of 0.52 (on the 0 to 1 scale) during illness, as compared to a mean score of 0.89 during the “post-illness” time period. When participants were split into two groups by minimum QWB score, the average scores were 0.39 and 0.64 for those in the ‘low’ and ‘high’ group. When split into three groups, the average minimum QWB scores for participants in the ‘low’, ‘medium’, and ‘high’ groups were 0.35, 0.52, and 0.67, respectively. There was no significant association of the minimum QWB score with age or sex.

### Visiting behavior

Approximately one-third (32%) of participants received visitors at some point during their illness. Females had visitors significantly more often than males (47% versus 19%; p = 0.02, Table 1). Of those participants who received visitors, most (48%) had one unique visitor and 70% of visitors came only once during the illness period. Those in the ‘low’ QWB group (lower health-related quality of life) were significantly more likely to receive multiple visitors compared to their ‘high’ QWB counterparts (82% versus 18%; p = 0.009; Table 1). Further, the QWB score was significantly associated with the relationship of the visitor (Fisher’s Exact test, p<0.001; Table 1). Seventy-seven percent of visitors received by those with ‘low’ QWB scores were family members, whereas visitors of those with ‘high’ QWB scores were more likely to be friends (62%) or be in the ‘other’ category (29%). Across all relationships, the majority of visitors were categorized by the ill participant as ‘routine visitors’ (visited at least once a week pre-illness) (87%), who knew that the participant was ill (87%), and were visiting for reasons related to the illness (80%) (Table 2). Additional details on the description of the reasons for visiting are presented in the supplemental materials (S1 Text). Of those individuals who had visitors before illness, 60% did not receive any visitors when ill, whereas 40% continued to receive visitors during illness. While the majority of participants who did not receive visitors before illness (70%) also did not receive visitors during illness, 30% did gain a visitor during their illness period.

**Table 1:** Differences in caregiving and visitor behaviors experienced by symptomatic DENV infected individuals in Iquitos, Peru, compared by sex, age, and Quality of Well-Being (QWB) score. Fisher’s Exact tests were performed for variables (1)whether caregiving was received, (2)if the person who helped was their housemate, (3) if the person who helped had their work negatively affected, (4) if visitors were received during the illness period, (5) whether one or two visitors were received, and (6) what the relationship of the visitor was (family/friend/other). The percent of each group (and the raw number of participants) that experienced a behavior is listed, as is the p-value for the Fisher’s Exact test. Participants’ minimum QWB score during illness were split into two equal groups, ‘Low’ and ‘High’, where those with ‘Low’ QWB scores had the worse health-related quality of life during illness. Variables (1),(4), and (5) were examined at the participant level, whereas (2),(3), and (6) were examined at the visitor/helper level. For variables (1)-(6) the overall N values in the first 2 columns (sex and age) were 67, 68, 68, 70, 23, and 85, respectively. As there were 2 participants without QWB scores available, overall N values in the QWB score column were 65, 65, 65, 68, 22, and 82, respectively.

	Sex			Age			QWB Score		
	Male	Female	p-value	Child <18 y.o.	Adult ≥18 y.o.	p-value	Low	High	p-value
**Someone Helped**	**94% (31)**	**85% (29)**	**0.4**	**98% (39)**	**78% (21)**	0.01*	**94% (31)**	**84% (27)**	**0.3**
**Helper Was Housemate**	**97% (33)**	**85% (29)**	**0.2**	**93% (41)**	**88% (21)**	**0.7**	**97% (33)**	**87% (27)**	**0.2**
**Helper’s Work Negatively Affected**	**18% (6)**	**35% (12)**	**0.2**	**18% (8)**	**42% (10)**	0.05*	**38% (13)**	**10% (3)**	0.01*
**Received Visitors**	**19% (7)**	**47% (16)**	0.02*	**29% (12)**	**38% (11)**	**0.6**	**32% (11)**	**32% (11)**	**1.0**
**Multiple Visitors**	**29% (2)**	**63% (10)**	**0.2**	**50% (6)**	**55% (6)**	**1.0**	**82% (9)**	**18% (2)**	0.009**
**Visitor Relationship**									
**Family**	**38% (8)**	**53% (34)**	**0.3**	**53% (26)**	**44% (16)**	**0.5**	**77% (37)**	**9% (3)**	<0.001***
**Friend**	**14% (3)**	**42% (27)**	0.03*	**45% (22)**	**22% (8)**	0.04*	**19% (9)**	**62% (21)**	<0.001***
**Other**	**48% (10)**	**5% (3)**	**<0.001** ***	**2% (1)**	**33% (12)**	<0.001***	**4% (2)**	**29% (10)**	0.003**

*p<0.05

** p<0.01

***p<0.001

**Table 2:** Characteristics of visitors received during illness by symptomatic DENV infected individuals in Iquitos, Peru. Data on visits received by ill individuals and the nature of these visits. Totals and categorized by if made by routine visitors. Whether the visitor knew of the illness, why they visited, and if they were classified as a ‘routine visitor’ were all reported by the ill participant.

		By Routine Visitor? (self-reported)	
	All Visits (n = 85)	Yes (n = 74)	No (n = 11)
Relationship of Visitor?			
Family	49.9%	55.4%	9.1%
Friend	35.3%	32.4%	54.6%
Other	15.3%	12.2%	36.4%
Visitor Knew of Illness?			
Yes	87.1%*	90.5%*	63.6%
Reason for Visit?			
Emotional Support	58.8%	66.2%	9.1%
Logistical Support	7.1%	6.8%	9.1%
Other Disease Support	11.8%	8.1%	36.4%
Not Related to Disease	20.0%*	16.2%*	45.5%
Routine Visitor?			
Yes	87.1%	---	---

*2 visits had missing values.

**Fig 1:**
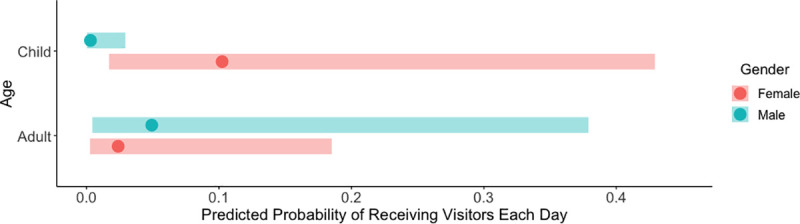
Predicted probability of receiving a visitor on each day of illness, based on a GLMM fitted to data of DENV infected individuals in Iquitos, Peru. Data are presented in terms of age group and sex. Error bars represent 95% Confidence Intervals.

According to the best-fit GLM, the sex of an ill individual was most associated with whether or not they received visitors during illness (S2 Table). Females were 3.7 times (95% CI: 1.2–10.7) more likely to than males to receive visitors during illness, with predicted probabilities of 50% and 20% for females and males, respectively. When examining the daily likelihood of receiving visitors during illness, the best-fit GLMM by AICc score included the interaction between age and sex (after accounting for participant ID as a random effect), where female children had the highest predicted probability of receiving visitors on any given day of illness (10%) and male children had the lowest daily probability (0.3%) (S3 Table and Fig 1). This model, however, was not significantly better than the GLMM including only sex and random effect of participant when looking at reduction in deviance (χ^2^ Analysis of Deviance, p = 0.08). In this model, females and males had predicted probabilities of 3.2% and 0.5% for receiving visitors on any given day of illness. Accounting for the specific day of illness did not have a significant effect on the fit of the model.

### Caregiving behavior

Of the 67 participants who provided data, 60 (90%) had someone help care for them during their illness. Of the seven individuals who did not receive help, five were adult females. Accordingly, children were significantly more likely than adults to receive help (98% vs. 78%; Fisher’s Exact test, p = 0.01) (Table 1). Further, the best-fit model for predicting whether or not caregiving was received included the age of the ill person, where the odds of receiving help was 12.3 times higher for children than adults (95% CI: 1.4–109.7) (S4 Table). There were no significant differences when comparing across age and sex combinations.

For those participants who had caregivers, caregivers most commonly were a relative who lived with them (91%) who helped for the length of the illness (94%). Only 11% of participants received help from two people. According to the best fit model, those who needed personal care help during illness were 5.6 times (95% CI: 1.0–31.5) more likely to receive help from two people compared to those who didn’t need personal care help (S5 Table). Among the people helping sick individuals, 28% had to take time off of work due to their caregiving behavior. It was more likely for work to be negatively affected when helping ill adults compared to ill children (42% vs 18%; Fisher’s Exact test, p = 0.05) and when helping those with low QWB scores compared to those with high QWB scores (39% vs 11%; Fisher’s Exact test, p = 0.01; Table 1). Indeed, the best-fit model for predicting whether a helper’s work would be negatively affected accounted for the participant’s minimum QWB score during illness, which was split into 3 groups of equal size (S6 Table). The predicted probability of having work adversely affected by helping those with the lowest QWB scores was 50%, as compared to a probability of 15% for those with medium QWB scores and 6% for those with high QWB scores. Accordingly, those helping individuals with the lowest QWB scores were more likely to have their work affected compared to those helping individuals with medium (OR: 0.18, 95% CI: 0.05–0.60) and high (OR: 0.07, 95% CI: 0.01–0.60) QWB scores. Additional details on the types of help given by caregivers are provided in the supplemental materials (S1 Text).

## Discussion

Socially-structured mobility patterns have been shown to play a significant role in determining dengue transmission patterns [7–11]. Similarly, recent studies have shown that symptomatic individuals have significant changes in their mobility patterns, which may have important effects on their contribution to onward transmission [13,14]. Here, we examined whether symptomatic DENV infection was associated with significant changes in the amount of time social contacts spent at the home of ill individuals.

We found that 32% of participants received visitors during their symptomatic illness, with females being significantly more likely to receive visitors. While the majority of visitors during illness were characterized as ‘routine’ pre-illness visitors, it was most common for them to visit only once during the illness period. Interestingly, visits were equally likely to occur on days 2–9 after symptom onset, rather than being more likely when symptoms started/were most intense. We also found no significant association between receiving visitors pre-illness (at day 0) and receiving visitors during illness. The majority of participants had the same visitor behaviors before and after illness, 53% with visitors at neither time point and 10% with visitors at both. There were, however, 15% of individuals who had visitors pre-illness, but not during illness (possibly due to social distancing behaviors) and 22% of individuals with no visitors pre-illness, but visitors during illness.

The majority of participants received help during their illness, although it was understandably more likely for children than adults (p = 0.01). Most often, caregivers were household members who helped for the length of the illness. The ‘housemates’ category accounts for adults helping adults (i.e., spouses) as well as parents helping sick children. Although a minority (28%) of caregivers reportedly had to take time off of work to help the ill individual (possibly affecting their overall mobility patterns), the negative impact on work was significantly associated with the symptomatic individual’s quality of life during illness. For those who had the lowest quality of life during illness (lowest QWB scores), 50% of helpers had their work adversely affected. Those with lower QWB scores were also more likely to have multiple helpers (and multiple visitors) compared to those higher QWB scores during illness.

Previous social support research found that most long-term assistance is received from family members, whereas short-term aid is mostly provided by friends and neighbors [49]. While our study was on a much shorter timescale, we saw similar trends with the majority of caregiving coming from relatives in the same household (91%) and lasting for the length of the illness (95%). Comparatively, the majority of visitors during illness were non-housemate family members and friends, 70% of whom only came once during the illness period. Further, while caregiving can have four different functions (emotional support, instrumental support, information, and appraisal), patients most often want their family and friends to provide emotional support during illness [34,50]. Our data supports this notion, with almost all caregivers helping to take care of the individual and 66% of visitors coming to give emotional support. Much of this previous research on caregiving behavior focused on helping individuals with non-communicable (e.g., cancer, heart disease) or sexually-transmitted (e.g., HIV) diseases [29,31,32], where increasing contact with ill individuals would not increase risk of infection. More recently, the social and cultural practices related to caregiving have been examined for highly transmissible diseases such as Ebola and COVID-19, where increased contact with ill individuals could increase risk of infection, underscoring the approach/avoidance conflict caregivers may face [33]. Indeed, control measures for Ebola focused on changing funerial practices to prevent risk for caregivers. In the case of dengue, and other VBDs where transmission is focused at the household level, caregiving and visitation of ill individuals increased time in the household and could cause an increase in exposure to infectious mosquitoes and a subsequent increase in infection risk. In order to minimize transmission risk for DENV, it is necessary not only to understand these disease-driven behavior changes but also to discern the social and cultural practices that drive them.

The impact of this novel dataset reaches beyond just dengue, allowing for empirical parameterization of social distancing and caregiving behaviors during the spread of other transmissible infections. Notably, our research elucidated caregiver and visitor behaviors that have not been accounted for in previous models of social contact mobility changes. The majority of caregivers were housemates of the ill individual, a sizable portion of whom had their work negatively affected due to their caregiving behaviors, particularly those caring for individuals with low quality of life. Prior models have not accounted for individuals spending more time at home when a housemate is ill, a behavior that could increase exposure to infectious mosquitoes. We saw a proportion of symptomatic individuals lose visitors during illness, a behavior that is commonly accounted for as social distancing [21,22,24]. We also saw, however, individuals gaining visitors during their illness period, a phenomenon that has not considered in previous network models examining mobility changes of social contacts. By accounting for the presence and prevalence of these opposing behaviors (social distancing, caregiving, and gaining visitors) in future transmission models, we can gain a more accurate understanding of the role of mobility change on epidemic spread.

Disease-driven mobility changes can have significant effects on the exposure of susceptible caregivers and visitors to infectious mosquitoes. In the case of DENV, these mobility changes may also have an effect on the onward transmission potential of caregivers and visitors with silent infections. Approximately 70% of DENV infections progress with mild or no symptoms, with many of these cases still being infectious to mosquitoes [42,51–54]. Further, those with symptomatic DENV are infectious to mosquitoes in the 1–2 days prior to symptom onset, i.e., the presymptomatic infectious period [55]. If social contacts acting as visitors/caregivers are silently infectious, changes in their behavior/mobility could impact their contact with susceptible mosquitoes and subsequent onward transmission of DENV. By acting as caregivers for housemates, presymptomatic and clinically inapparent infected individuals would be spending more time at home and less time in other places, in effect changing their mobility the same way as symptomatic individuals. The effect of this for onward transmission will depend on the distribution of mosquitoes in their home compared to the other places they would otherwise frequent [15]. Alternatively, routine visitors who socially distance themselves (i.e., stop visiting during illness) could experience changes in their potential to transmit DENV depending on where they spend this extra time. The high proportion of silently infectious people in the population allows the mobility changes of social contacts to impact DENV spread not only through virus exposure, but also through potential onward transmission. These impacts of social distancing and caregiving behaviors on transmission should, therefore, be examined for other infections where inapparent cases and presymptomatic infectiousness are common, such as COVID-19, for which social distancing is currently playing a significant role [56,57].

One limitation of our study was the reliance on participant recall, which can be subject to recall bias or respondent fatigue; however, the RMS survey we used was previously tested in Iquitos and found to be superior to GPS data-loggers in collecting data on activity space [43]. Moreover, it was applied daily–and when one is ill, a limited number of places are visited. Our study was also constrained by the small sample size, which could lead to spurious correlations or result in valid associations lacking statistical significance. In addition, our baseline visitor data were only recorded for the three days prior to the first survey, which may have excluded data on routine visitors who visit once every one or two weeks. As infected participants were not always captured immediately after symptom onset, the first survey likely collected data on visitors coming before and after symptom onset, which may have skewed our ‘baseline’ values. Our analysis of the impact of caregiving on mobility relied on the metric of whether an individual had to take time off of work. It is possible that caregivers who didn’t have their work affected still had significant changes to their mobility, especially given the high proportion of participants who classified their work schedule as ‘flexible’. Our study focused on the social visits a symptomatic individual received at their home; however, individuals may also have received customers to a home-based business (i.e., a store operating at the front of the home), as is common in Iquitos. These economically-driven household visits may play a role in transmission, particularly if the business is kept open when the ill individual is infectious or there are infected mosquitoes in the space. Although our study is, to the best of our knowledge, the first to examine the mobility behavior of social contacts during symptomatic dengue illness, our findings demonstrate that the effects of dengue illnesses on human behavior are potentially farther-reaching than was previously considered. Additional research is needed to examine a more extensive group of individuals, more occasional visitors, and more minor mobility changes.

Our results show that symptomatic dengue is significantly associated with changes in the mobility patterns of not only ill individuals, but also their social contacts. These mobility changes could have significant impacts on a susceptible contact’s exposure to virus or a presymptomatic/clinically inapparent contact’s contribution to onward transmission. We found that the age and gender of a symptomatic individual can predict whether they receive visitors or caregivers, whereas their quality of life during illness can determine if a social contact’s behavior change significantly affects their mobility patterns. Using these associations, we can more accurately account for the dynamic nature of social contacts during a symptomatic DENV infection, allowing for improved disease transmission models and more efficient design and evaluation of disease prevention strategies.

## Supporting information

S1 Checklist STROBE Checklist. Checklist used for reporting observational studies.
(DOC)

S1 Text What help was received. (DOCX)

S1 Table Description of surveys. Provides descriptions of each survey and questions of interest on the survey, as well as the time point when each survey was administered to individuals, how the data were aggregated for analysis, the number of respondents total, and the number of respondents who also have data on ‘Expenses’ and ‘Daily Visitors’.
(PDF)

S2 Table Comparison of logistic GLMs for whether or not visitors were received. Amount of deviance explained (%), degrees of freedom (df), change in AICc compared to best fit model (ΔAICc), and model weight are provided for each model. The best-fit model is highlighted in red.
(PDF)

S3 Table Comparison of logistic GLMMs for whether or not visitors were received for each day of illness. Amount of deviance explained (%), degrees of freedom (df), change in AICc compared to best fit model (ΔAICc), and model weight are provided for each model. The best-fit model is highlighted in red.
(PDF)

S4 Table Comparison of logistic GLMs for whether or not caregiving was received. Amount of deviance explained (%), degrees of freedom (df), change in AICc compared to best fit model (ΔAICc), and model weight are provided for each model. The best-fit model is highlighted in red.
(PDF)

S5 Table Comparison of logistic GLMs for whether the ill individual had one or two caregivers. Amount of deviance explained (%), degrees of freedom (df), change in AICc compared to best fit model (ΔAICc), and model weight are provided for each model. The best-fit model is highlighted in red.
(PDF)

S6 Table Results from likelihood ratio tests between pairs of logistic GLMs with various explanatory variables and response variable of whether or not the helper’s work was affected. Amount of deviance explained (%), degrees of freedom (df), change in AICc compared to best fit model (ΔAICc), and model weight are provided for each model. The best-fit model is highlighted in red.
(PDF)

S7 Table Differences in reason visitors were received by symptomatic DENV infected individuals in Iquitos, Peru, compared by sex, age, QWB score, and routineness of the visitor. Fisher’s Exact tests were performed for whether or not visitors came to give emotional support, logistic support, another type of disease-related reason, or for a reason unrelated to the disease. The percent of each group (and the raw number of participants) were given for each visitation reason listed, as is the p-value for the Fisher’s Exact test. (*p<0.05, ** p<0.01, ***p<0.001).
(PDF)

S8 Table Frequency of in-home caregiving behaviors provided to symptomatic DENV infected individuals in Iquitos, Peru. Given as the number and percent of helpers (out of 67).
(PDF)

S9 Table Differences in types of caregiving received by symptomatic DENV infected individuals in Iquitos, Peru, compared by sex, age, and QWB score. Fisher’s Exact tests were performed for whether the type of help was taking care of the individual, helping around the house, or helping with money and buying things. The percent of each group (and the raw number of participants) that received each type of help is listed, as is the p-value for the Fisher’s Exact test. (*p<0.05, ** p<0.01, ***p<0.001).
(PDF)

S10 Table Comparison of logistic GLMs for whether or not help was received in the form of money and things. Amount of deviance explained (%), degrees of freedom (df), change in AICc compared to best fit model (ΔAICc), and model weight are provided for each model. The best-fit model is highlighted in red.
(PDF)

S11 Table Comparison of logistic GLMs for whether or not help was received around the house. Amount of deviance explained (%), degrees of freedom (df), change in AICc compared to best fit model (ΔAICc), and model weight are provided for each model. The best-fit model is highlighted in red.
(PDF)

S1 Fig Predicted probability of receiving money or things as a form of caregiving during symptomatic DENV infection, based on a GLM fitted to data from Iquitos, Peru. Given in terms of number of housemates and whether the individual reported needed personal care during illness. Error bars represent 95% Confidence Intervals.
(TIF)
