# Supplementary material for: The impact of dengue illness on social distancing and caregiving behavior
Source: PLoS Negl Trop Dis. 2021 Jul 19;15(7):e0009614. doi: 10.1371/journal.pntd.0009614 (PMC8354465; doi:10.1371/journal.pntd.0009614)
Supplement: S1 Text — (DOCX) [file pntd.0009614.s002.docx]

**S1 Text. What Help Was Received.**

The majority (80.5%) of visits made to symptomatic dengue individuals were for reasons related to the illness. However, this was significantly different based on age, where adults were more likely to receive visitors for disease-related emotional support (75.0% vs. 48.9%) (Fisher Exact test, p=0.02) and children were more likely than adults to receive visitors for reasons unrelated to their disease (29.8% vs. 8.3%) (Fisher Exact test, p=0.03) (S7 Table). The reason for visiting was also associated with whether or not the visitor was ‘routine’. ‘Routine’ visitors were significantly more likely to provide emotional support (68.1% vs. 9.1%) (Fisher Exact test, p<0.001), whereas ‘non-routine’ visitors were more likely to visit for a non-emotional, non-logistic, disease-related reason (36.4% vs. 8.3%) (Fisher Exact test, p=0.02) and more likely to visit for reasons unrelated to the disease (45.5% vs. 16.7%) (Fisher Exact test, p=0.04) (S7 Table). There were no significant differences in why visitors were received by those with ‘low’ and ‘high’ QWB scores during illness.

When symptomatic individuals received help from caregivers, the most common way of helping was by taking care of them (95.6%), with 47.1% of caregivers helping around the house (taking care of children, cooking, cleaning) and 38.2% helping by buying things for the sick individual or giving them money (S8 Table). The largest proportion of people (34.3%) helped only with taking care of the sick person; however, 20.9% of people helped in all three ways (S8 Table). There were no significant differences in the type of help provided based on QWB score, age, or gender, although getting help around the house was quite common for adult males (75.0%) compared to adult females (41.7%) (Fisher’s Exact test, p=0.2) and children (40.9%) (Fisher’s Exact test, p=0.08) (S9 Table).

The best-fit model for whether or not an individual received help in the form of money or things accounted for how many house members they had (less than or greater than median of 8) and whether they needed personal care help during their illness (S10 Table). Individuals who needed help with personal care and had a large number of housemates had a higher predicted probability of receiving money and things than those who didn’t need personal care help and/or live with fewer housemates (S1 Fig). Accordingly, those who needed personal care help during illness were 18.2 times (95% CI: 2.0 – 166.8) more likely to receive money and things than those who didn’t and those with a large number of housemates were 3.6 times (95% CI: 1.1 – 12.1) more likely to receive money and things than those with a small number of housemates. The best-fit model by AICc score for predicting whether or not help was received around the house included whether or not daily help was needed; however, this was not significantly better than the null model when looking at reduction in deviance (χ^2^ Analysis of Deviance, p=0.11) (S11 Table).

There was no significant difference in the way the caregivers helped or the reason for a visitor’s visit when looking at the 20 individuals who received both visitors and caregivers during illness.
