# Supplementary material for: The impact of dengue illness on social distancing and caregiving behavior
Source: PLoS Negl Trop Dis. 2021 Jul 19;15(7):e0009614. doi: 10.1371/journal.pntd.0009614 (PMC8354465; doi:10.1371/journal.pntd.0009614)
Supplement: S1 Table — Provides descriptions of each survey and questions of interest on the survey, as well as the time point when each survey was administered to individuals, how the data were aggregated for analysis, the number of respondents total, and the number of respondents who also have data on ‘Expenses’ and ‘Daily Visitors’. (PDF) [file pntd.0009614.s003.pdf]

Questions of Interest	When Administered (days after blood test)	Survey Description	Number of Respondents Total:	Survey Name
• Did anyone help take care of you during illness?<br>• How many people?<br>• What was their relationship to you?<br>• How did they help you?<br>• Was their work affected by helping you?	Day 7<br>Day 30	To look at the direct costs (money spent on medication, etc.) and indirect (lost income due to absence at work) costs incurred during illness for the ill individual and possible caretakers.	67 (67)	Expenses
• Did you have visitors?<br>• What was their relationship to you?<br>• Why were they visiting?<br>• Did they know you were ill at the time of the visit?<br>• Do these individuals routinely visit you (without illness)?	Days 1-7	To determine whether visitors were received at an individual's home. (Part of a larger retrospective movement survey modified to identify locations visited, time spent at home, and visitors received in the previous 24 hours of illness.)	71 (67)	Daily Visitors subsection of the Daily Retrospective Movement Survey (DRMS)
• Did you *need help* with daily activities?<br>• Did you *need help* with personal care activities?<br>• Weighted well-being score	Day 2-3<br>Day 7<br>Day 30	To measure an individual's quality of life as a well-being score ranging between 0.0 (death) and 1.0 (asymptomatic and fully-functioning).	70 (67)	Quality of Well-Being (QWB)
