# Supplementary material for: The impact of dengue illness on social distancing and caregiving behavior
Source: PLoS Negl Trop Dis. 2021 Jul 19;15(7):e0009614. doi: 10.1371/journal.pntd.0009614 (PMC8354465; doi:10.1371/journal.pntd.0009614)
Supplement: S2 Table — Amount of deviance explained (%), degrees of freedom (df), change in AICc compared to best fit model (ΔAICc), and model weight are provided for each model. The best-fit model is highlighted in red. (PDF) [file pntd.0009614.s004.pdf]

Predictor Variable(s)	Deviance	df	AICc	Δ AICc	Weight
Intercept		1	87.7	3.8	0.080
Sex	5.91	2	83.9	0.0	0.531
Age (<18)	0.45	2	89.4	5.5	0.035
Sex * Age	7.68	4	86.6	2.7	0.139
Number Housemates (<8)	0.10	2	89.7	5.8	0.029
Minimum QWB Score	0.72	2	89.1	5.2	0.040
Minimum QWB Score (low/high)	0.00	2	89.8	5.9	0.028
Minimum QWB Score (low/med/high)	0.90	3	91.1	7.2	0.015
Needed Help with Personal Care (QWB)	0.03	2	89.8	5.9	0.028
Needed Help with Daily Activities (QWB)	0.50	2	89.3	5.4	0.035
Visitors Received on Original (Day 0) survey	0.79	2	89.0	5.1	0.041
