# Supplementary material for: The impact of dengue illness on social distancing and caregiving behavior
Source: PLoS Negl Trop Dis. 2021 Jul 19;15(7):e0009614. doi: 10.1371/journal.pntd.0009614 (PMC8354465; doi:10.1371/journal.pntd.0009614)
Supplement: S3 Table — Amount of deviance explained (%), degrees of freedom (df), change in AICc compared to best fit model (ΔAICc), and model weight are provided for each model. The best-fit model is highlighted in red. (PDF) [file pntd.0009614.s005.pdf]

Predictor Variable(s)	Deviance	df	AICc	Δ AICc	Weight
Intercept		2	228.5	2.0	0.122
Day of Illness	4.38	11	242.8	16.4	<0.001
Sex	3.17	3	227.4	0.9	0.215
Age (<18)	0.44	3	230.1	3.6	0.055
Sex * Age	8.17	5	226.5	0.0	0.337
Number Housemates (<8)	0.02	3	230.5	4.1	0.044
Minimum QWB Score	0.54	3	230.0	3.5	0.058
Minimum QWB Score (low/high)	0.14	3	230.4	3.9	0.047
Minimum QWB Score (low/med/high)	0.81	4	231.8	5.3	0.024
Needed Help with Personal Care (QWB)	0.003	3	230.5	4.1	0.044
Needed Help with Daily Activities (QWB)	0.37	3	230.2	3.7	0.053
