# Supplementary material for: The impact of dengue illness on social distancing and caregiving behavior
Source: PLoS Negl Trop Dis. 2021 Jul 19;15(7):e0009614. doi: 10.1371/journal.pntd.0009614 (PMC8354465; doi:10.1371/journal.pntd.0009614)
Supplement: S4 Table — Amount of deviance explained (%), degrees of freedom (df), change in AICc compared to best fit model (ΔAICc), and model weight are provided for each model. The best-fit model is highlighted in red. (PDF) [file pntd.0009614.s006.pdf]

Predictor Variable(s)	Deviance	df	AICc	Δ AICc	Weight
Intercept		1	46.5	5.4	0.022
Sex	1.59	2	47.0	5.9	0.017
Age (<18)	7.51	2	41.1	0.0	0.331
Sex * Age	11.33	4	41.8	0.7	0.239
Number Housemates (<8)	3.51	2	45.1	4.0	0.045
Minimum QWB Score	6.20	2	42.4	1.3	0.173
Minimum QWB Score (low/high)	1.59	2	47.0	5.9	0.017
Minimum QWB Score (low/med/high)	7.96	3	42.9	1.8	0.138
Needed Help with Personal Care (QWB)	0.007	2	48.6	7.5	0.008
Needed Help with Daily Activities (QWB)	0.38	2	48.2	7.1	0.009
