# Supplementary material for: The impact of dengue illness on social distancing and caregiving behavior
Source: PLoS Negl Trop Dis. 2021 Jul 19;15(7):e0009614. doi: 10.1371/journal.pntd.0009614 (PMC8354465; doi:10.1371/journal.pntd.0009614)
Supplement: S5 Table — Amount of deviance explained (%), degrees of freedom (df), change in AICc compared to best fit model (ΔAICc), and model weight are provided for each model. The best-fit model is highlighted in red. (PDF) [file pntd.0009614.s007.pdf]

Predictor Variable(s)	Deviance	df	AICc	Δ AICc	Weight
Intercept		1	44.8	1.4	0.147
Sex	0.36	2	46.6	3.2	0.060
Age (<18)	0.06	2	46.9	3.5	0.052
Sex * Age	5.60	4	45.9	2.5	0.086
Number Housemates (<8)	0.13	2	46.8	3.4	0.053
Minimum QWB Score	0.40	2	46.5	3.2	0.061
Minimum QWB Score (low/high)	0.36	2	46.6	3.2	0.060
Minimum QWB Score (low/med/high)	0.81	3	48.4	5.0	0.025
Needed Help with Personal Care (QWB)	3.56	2	43.4	0.0	0.297
Needed Help with Daily Activities (QWB)	2.30	2	44.6	1.3	0.159
