# Supplementary material for: The impact of dengue illness on social distancing and caregiving behavior
Source: PLoS Negl Trop Dis. 2021 Jul 19;15(7):e0009614. doi: 10.1371/journal.pntd.0009614 (PMC8354465; doi:10.1371/journal.pntd.0009614)
Supplement: S6 Table — Amount of deviance explained (%), degrees of freedom (df), change in AICc compared to best fit model (ΔAICc), and model weight are provided for each model. The best-fit model is highlighted in red. (PDF) [file pntd.0009614.s008.pdf]

Predictor Variable(s)	Deviance	df	AICc	Δ AICc	Weight
Intercept		1	68.4	7.0	0.015
Sex	1.47	2	69.1	7.7	0.011
Age (<18)	3.73	2	66.8	5.5	0.034
Sex * Age	5.43	4	69.6	8.3	0.008
Number Housemates (<8)	0.27	2	70.3	8.9	0.006
Minimum QWB Score	7.90	2	62.6	1.3	0.273
Minimum QWB Score (low/high)	6.09	2	64.4	3.1	0.111
Minimum QWB Score (low/med/high)	11.42	3	61.3	0.0	0.523
Needed Help with Personal Care (QWB)	1.75	2	68.8	7.4	0.013
Needed Help with Daily Activities (QWB)	0.08	2	70.5	9.2	0.005
