# Supplementary material for: The impact of dengue illness on social distancing and caregiving behavior
Source: PLoS Negl Trop Dis. 2021 Jul 19;15(7):e0009614. doi: 10.1371/journal.pntd.0009614 (PMC8354465; doi:10.1371/journal.pntd.0009614)
Supplement: S7 Table — Fisher’s Exact tests were performed for whether or not visitors came to give emotional support, logistic support, another type of disease-related reason, or for a reason unrelated to the disease. The percent of each group (and the raw number of participants) were given for each visitation reason listed, as is the p-value for the Fisher’s Exact test. (*p<0.05, ** p<0.01, ***p<0.001). (PDF) [file pntd.0009614.s009.pdf]

| | Sex | | | Age | | | QWB Score | | | Visitor 'Routineness' | | |
|---|---|---|---|---|---|---|---|---|---|---|---|---|
| | Male | Female | p-value | Child | Adult | p-value | Low | High | p-value | Routine | Non-Routine | p-value |
| Visit for Emotional Support | 71% (15) | 56% (35) | 0.3 | 49% (23) | 75% (27) | 0.02* | 62% (29) | 58% (19) | 0.8 | 68% (49) | 9% (1) | <0.001 *** |
| Visit for Logistic Support | 5% (1) | 8% (5) | 1.0 | 11% (5) | 3% (1) | 0.2 | 13% (6) | 0% (0) | 0.04* | 7% (5) | 9% (1) | 0.6 |
| Visit for Other Disease Reason | 5% (1) | 14% (9) | 0.4 | 11% (5) | 14% (5) | 0.7 | 9% (4) | 15% (5) | 0.5 | 8% (6) | 36% (4) | 0.02* |
| Visit Unrelated to Disease | 19% (4) | 21% (13) | 1.0 | 30% (14) | 8% (3) | 0.03* | 17% (8) | 27% (9) | 0.3 | 17% (12) | 45% (5) | 0.04* |
