# Supplementary material for: The impact of dengue illness on social distancing and caregiving behavior
Source: PLoS Negl Trop Dis. 2021 Jul 19;15(7):e0009614. doi: 10.1371/journal.pntd.0009614 (PMC8354465; doi:10.1371/journal.pntd.0009614)
Supplement: S8 Table — Given as the number and percent of helpers (out of 67). (PDF) [file pntd.0009614.s010.pdf]

Help Take Care of Person	Help Around House*	Help with Money/Buying Things	Number of Helpers
Yes	Yes	Yes	14 (20.9%)
Yes	Yes	No	17 (25.4%)
Yes	No	Yes	11 (16.4%)
Yes	No	No	23 (34.3%)
No	Yes	Yes	0 (0%)
No	Yes	No	0 (0%)
No	No	Yes	2 (3.0%)
No	No	No	0 (0%)

* Includes help with taking care of kids, cooking, and cleaning
