# Supplementary material for: The impact of dengue illness on social distancing and caregiving behavior
Source: PLoS Negl Trop Dis. 2021 Jul 19;15(7):e0009614. doi: 10.1371/journal.pntd.0009614 (PMC8354465; doi:10.1371/journal.pntd.0009614)
Supplement: S9 Table — Fisher’s Exact tests were performed for whether the type of help was taking care of the individual, helping around the house, or helping with money and buying things. The percent of each group (and the raw number of participants) that received each type of help is listed, as is the p-value for the Fisher’s Exact test. (*p<0.05, ** p<0.01, ***p<0.001). (PDF) [file pntd.0009614.s011.pdf]

	Sex			Age			QWB Score		
	Male	Female	p-value	Child	Adult	p-value	Low	High	p-value
Helped Take Care of Me	97%(33)	94%(32)	1.0	95%(42)	96%(23)	1.0	97%(33)	94%(29)	0.6
Helped Around House	53%(18)	41%(14)	0.5	41%(18)	58%(14)	0.2	41%(14)	58%(18)	0.2
Helped with Money/Things	35%(12)	41%(14)	0.8	36%(16)	42%(10)	0.8	41%(1$)	39%(12)	1.0
