# Supplementary material for: The impact of dengue illness on social distancing and caregiving behavior
Source: PLoS Negl Trop Dis. 2021 Jul 19;15(7):e0009614. doi: 10.1371/journal.pntd.0009614 (PMC8354465; doi:10.1371/journal.pntd.0009614)
Supplement: S10 Table — Amount of deviance explained (%), degrees of freedom (df), change in AICc compared to best fit model (ΔAICc), and model weight are provided for each model. The best-fit model is highlighted in red. (PDF) [file pntd.0009614.s012.pdf]

Predictor Variable(s)	Deviance	df	AICc	Δ AICc	Weight
Intercept		1	81.4	9.7	0.006
Sex	00.52	2	83.0	11.3	0.002
Age (<18)	1.04	2	82.5	10.8	0.003
Sex * Age	1.61	4	86.5	14.8	0.0004
Number Housemates (<8)	3.55	2	80.0	8.3	0.011
Minimum QWB Score	0.64	2	82.9	11.2	0.003
Minimum QWB Score (low/high)	0.12	2	83.4	11.7	0.002
Minimum QWB Score (low/med/high)	1.38	3	84.4	12.7	0.001
Needed Help with Personal Care (QWB)	9.76	2	73.8	2.1	0.251
Needed Help with Daily Activities (QWB)	1.40	2	82.1	10.5	0.004
Number Housemates (<8) + Needed Help with Personal Care (QWB)	14.09	3	71.7	0.0	0.717
