# Supplementary material for: The impact of dengue illness on social distancing and caregiving behavior
Source: PLoS Negl Trop Dis. 2021 Jul 19;15(7):e0009614. doi: 10.1371/journal.pntd.0009614 (PMC8354465; doi:10.1371/journal.pntd.0009614)
Supplement: S11 Table — Amount of deviance explained (%), degrees of freedom (df), change in AICc compared to best fit model (ΔAICc), and model weight are provided for each model. The best-fit model is highlighted in red. (PDF) [file pntd.0009614.s013.pdf]

Predictor Variable(s)	Deviance	df	AICc	Δ AICc	Weight
Intercept		1	82.5	0.4	0.174
Sex	0.69	2	84.6	2.5	0.061
Age (<18)	1.97	2	82.7	0.6	0.159
Sex * Age	2.41	4	86.8	4.7	0.020
Number Housemates (<8)	0.31	2	84.3	2.2	0.069
Minimum QWB Score	0.19	2	84.4	2.3	0.065
Minimum QWB Score (low/high)	1.74	2	82.9	0.8	0.142
Minimum QWB Score (low/med/high)	1.24	3	85.6	3.5	0.036
Needed Help with Personal Care (QWB)	0.13	2	84.5	2.4	0.063
Needed Help with Daily Activities (QWB)	2.53	2	82.1	0.0	0.210
