# Supplementary figures and images for: The impact of dengue illness on social distancing and caregiving behavior
Source: PLoS Negl Trop Dis. 2021 Jul 19;15(7):e0009614. doi: 10.1371/journal.pntd.0009614 (PMC8354465; doi:10.1371/journal.pntd.0009614)

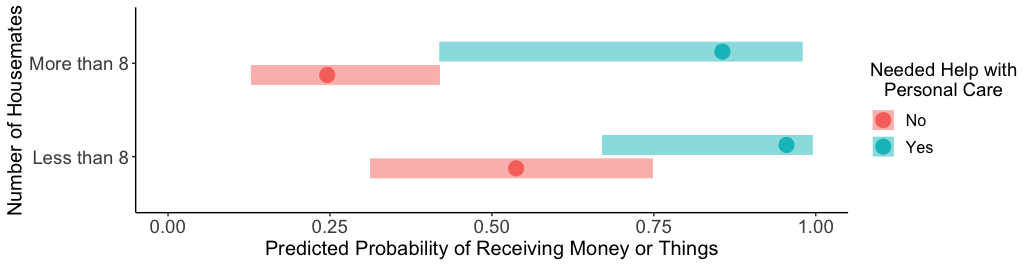

Supplement: S1 Fig — Given in terms of number of housemates and whether the individual reported needed personal care during illness. Error bars represent 95% Confidence Intervals. (TIF) [file pntd.0009614.s014.tif]
